# Influence of Behavioral and Sociodemographic Factors on Dental Caries in Mexican Children

**DOI:** 10.3390/pediatric17020040

**Published:** 2025-03-26

**Authors:** Ángel Pérez-Reyes, Julieta Sarai Becerra-Ruiz, Juan Manuel Guzmán-Flores

**Affiliations:** 1 División de Ciencias Biomédicas, Centro Universitario de Los Altos, Universidad de Guadalajara, Tepatitlán de Morelos C.P. 47620, Jalisco, Mexico; angel.perez1047@alumnos.udg.mx; 2 Departamento de Clínicas, Centro Universitario de Los Altos, Universidad de Guadalajara, Tepatitlán de Morelos C.P. 47620, Jalisco, Mexico; julieta.becerra@academicos.udg.mx; 3 Departamento de Ciencias de la Salud, Centro Universitario de Los Altos, Universidad de Guadalajara, Tepatitlán de Morelos C.P. 47620, Jalisco, Mexico

**Keywords:** caries, habits, feeding behavior, children, Mexican

## Abstract

Background: In Mexico, tooth decay is common among children and is on the rise. A strong relationship exists between behavioral, socioeconomic, demographic, and biological factors and the severity and development of dental caries. Objective: The present study analyzed the risk factors contributing to dental caries in a Mexican pediatric population. Materials and Methods: This study employed a cross-sectional design. One hundred fifty-one children were evaluated after providing their assent and the informed consent of their legal guardians. The study subjects recruited underwent dental assessments, dietary habits, and physical activity examinations. Results: Only 36 children were classified as overweight or obese, while 121 children had a high quality of life related to oral health. Most of the children showed deficient eating and physical activity habits. Additionally, nearly half displayed severe dental carie lesions. The occurrence of caries was linked to both age and tooth brushing habits. The median of teeth with initial caries lesions was higher among female children. Furthermore, parental education was associated with the presence of fluorosis and the frequency of tooth brushing. Conclusion: Our study revealed deficient eating habits and a high prevalence of dental caries among children aged 6–12. Additionally, parents and guardians demonstrated insufficient knowledge about general oral health issues, the risk factors for caries, and their potential consequences, which means a need for both preventive and corrective treatments. This highlights the need for strategies focused on improving oral health care and prevention.

## 1. Introduction

Dental caries is a non-communicable and reversible disease that occurs when bacterial plaque forms on the tooth surface and converts sugars in food and beverages into acids that demineralize tooth enamel over time [1]. It is estimated that 530 million children worldwide suffer from dental caries in the primary dentition [1]. Over the last 30 years (1990–2019), the estimated case numbers of major oral diseases (caries of deciduous) in the region of the Americas grew by more than 151 million, a 48% increase [2]. In Mexico, there is a 45.8% prevalence of childhood caries in primary dentition [1]. A study in Tepatitlán, Jalisco, found a high prevalence of caries [3]. The caries diagnosis in epidemiological studies is performed using the “International Caries Detection and Assessment System” (ICDAS) [4].

The etiology of caries is complex and inconclusive since several risk factors are related. For example, socioeconomic, demographic, biological, local, systemic, genetic, environmental, level of cariogenic microorganisms, immune response, dental anatomy, diet, general health, and behavioral characteristics are directly involved in the development of caries lesions [5,6,7].

There is controversy about which of the multiple factors involved in caries may impact its occurrence and development and how it can be measured; for example, several proteins that are part of the immune system are differentially expressed in the saliva of children with caries, and the metabolic pathways of these proteins are related to folate, selenium, and vitamin B12 metabolism [8], suggesting a relationship with specific nutrients in the diet.

On the other hand, there are not sufficiently designed and validated tools that accurately and reliably evaluate the cause of this complex disease [9]. However, the tools used in this study have been validated and adapted for the Mexican population and are reliable in obtaining the information required for operationalization and interpretation.

From childhood to old age, the relationship between economic status (income, occupation, and educational level), prevalence, and severity of oral diseases is very clear and persistent [10]. It has been observed that there is a close relationship between a high prevalence of caries in children and a low family socioeconomic status [11,12], in addition to parental occupation [13]; also, these families have a diet rich in sugars and fats [11,14]. In Mexican families with a low income, there is a more significant presence of caries [15,16], maybe due to the families’ socioeconomic reality where carbohydrate-based foods are more accessible and to the fact that schoolchildren have multiple snacks during school hours, where they spend most of the day [9].

Instead, oral hygiene at an early age is significantly associated with a low prevalence of caries [12] since regular toothbrushing represents a lower risk for caries development [17,18,19].

On the other hand, a consequence of the lack of physical activity in the first years of life is an increased risk of being overweight, obesity, and tooth decay. Lack of physical activity could lead to an increase in television viewing time [20] and, consequently, an increase in the consumption of unhealthy foods [21], such as sugars; this is also related to the development of caries. Thus, caries share risk factors with prevalent diseases such as obesity and diabetes [11]. Studies confirm that overweight and obese children have a higher risk of caries lesions compared to normal-weight children [22,23,24].

Lifestyle changes, mainly poor eating habits and poor oral hygiene, as well as the genetic makeup of the Mexican pediatric population [25], have led to an accelerated and continuous increase in the prevalence of caries. Affliction from this disease has an impact on both the quality of life and the lifestyle of the children who suffer from it, in addition to eventually affecting the economy, both for the health institutions and for the families [26].

Nowadays, several non-invasive methods are available that could intercept, control, slow down, or arrest carious lesions, and preventive measures involve standard oral home care and regular oral hygiene, dental floss, dietary counseling, proper exposure to fluorides, regular control dental visits, and mechanical removal of dental plaque, but these strategies have limitations, such as challenges with compliance, the rapid recolonization of bacteria, the behavioral and socioeconomic barriers to dietary modifications, and their inability to address the underlying microbial imbalance driving caries development [27,28]. Currently, adjuvant therapies, such as curcumin, have emerged, since curcumin affects both host carious processes and streptococcus mutans [29].

It is hypothesized that behavioral and demographic risk factors are associated with caries development in the Mexican pediatric population. Identifying risk factors involved in caries development will allow us to define the disease’s prognosis and the role of the interactions between these factors, mainly to understand and minimize the impact that this pathology generates in the Mexican pediatric population.

This research aims to analyze the contribution of risk factors involved in caries severity in a Mexican pediatric population.

## 2. Materials and Methods

### 2.1. Participants and Procedures

The present work was approved by the research and ethics (CEI-01-2023-01) and biosafety (CBIO-01-2023-01) local committees. The parents and pediatric patients issued, signed, and accepted a letter of consent and assent. All the recommendations presented by the committees were attended to, and the indications established in the Official Standards and standard operating procedures, including personal protective equipment, were followed. The sample size was calculated with an expected prevalence of 0.95 (95%), a confidence level of (1 − α) 95% (Z = 1.96), and a precision (d) of 0.05 (5%). The expected prevalence was obtained from a previous study conducted in the same region [23].

Dental analysis data and dietary and physical activity considerations were collected at the Comprehensive Medical Care Center, which belongs to the Centro Universitario de Los Altos of the Universidad of Guadalajara.

A validated and adapted instrument for the Mexican population concerning “Quality of life related to oral health” called the “Early Childhood Oral Health Impact Scale” (ECOHIS) was applied [30]. The ECOHIS was answered by the parent/guardian to assess the impact of oral health problems on the quality of life of children and their families. It comprises six possible answers for each question on Likert-type frequency scales [31].

The pediatric patients were administered a “Healthy Lifestyle Habits Questionnaire on Eating and Physical Activity” (CHVSAAF), which was designed for schoolchildren up to 12 years of age; identifying lifestyle habits of two sections, which records the frequency of consumption of certain foods, and physical activity and inactivity, which records rest and physical activation activities. According to the total score obtained, the pediatric patients’ eating and physical activity habits were classified as deficient (the child’s lifestyle poses many risk factors for health damage) and adequate (lifestyle and habits effectively influence optimal health); this classification is of most significant importance for intervention programs that promote improvements in health and quality of life [32].

The determination of obesity or overweight was carried out using a digital scale (Trade: Tanita; model: BC-730F) and considering the Body Mass Index (BMI), in this case, for subjects aged 5–19 years and according to the World Health Organization (WHO). Overweight is a BMI for age more than one standard deviation above the median established in the WHO Child Growth Standards, and obesity is more significant than two standard deviations above the median established in the WHO Child Growth Standards [33].

The diagnosis of caries in mixed dentition was made according to the ICDAS system and performed by calibrated pediatric dentists using the WHO rounded-end probe. The diagnosis was defined as a healthy stage “ICDAS 0”, initial stage “ICDAS 1 and 2”, moderate “ICDAS 3 and 4”, and severe “ICDAS 5 and 6”.

### 2.2. Demographic Data

The study population consisted of 151 children aged 6–12 years; out of 151 children examined, 66 (43.7%) were male, and 85 (56.3%) were female. The age of the population was 8.7 ± 1.7 (mean plus or minus standard deviation), and only 36 (23.8%) children were overweight or obese. ECOHIS found that 121 children showed a low oral health impact in childhood, which translates into a high quality of life regarding oral health. Concerning the parents’ education, 31 had completed high school, and only 9.9% had a bachelor’s degree or higher.

The vast majority (85.2%) of the children recruited showed poor dietary and physical activity habits, and almost half (43.7%) presented a severe caries lesion. Likewise, most children had at least one caries lesion (84.1%); only 20.5% showed (molar incisor hypomineralization) MIH and 6.0% fluorosis. Regarding oral hygiene habits, almost half of the children brush their teeth more than once daily, although only 6 (4%) use dental floss (Table 1).

### 2.3. Statistical Analysis

Descriptive statistics of qualitative variables are presented as simple frequency and percentage. Quantitative variables are presented as mean, standard deviation, or median and interquartile range. After normality analysis, a univariate analysis was performed for inferential statistics using the chi-square test, T student, ANOVA, or their corresponding non-parametric tests.

## 3. Results

Table 2 shows some variables compared to the presence or absence of caries lesions. There is a higher number of children with caries (*n* = 127) than without caries (*n* = 24). Regarding age, there is a statistical difference (*p* = 0.027) between the age groups and the presence or absence of caries lesions. It is worth mentioning that all the 10-year-old children showed a caries lesion. Regardless of the frequency of tooth brushing, there is a higher percentage of children with caries lesions in the pediatric population studied. However, a statistical difference exists between those who brush 0–1 times/day and those who brush twice or more times/day (*p* = 0.026). No significant statistical differences were found related to other variables, as shown in Table 2.

Interestingly, regarding caries severity, there were no differences in the pediatric population studied concerning the variables analyzed; the data are shown in Table 3.

Regarding sex, females showed a higher median of teeth with initial caries lesions (Table 4).

Finally, when we analyzed the oral characteristics concerning parents’ education, we found a statistically significant difference between fluorosis and brushing frequency (*p* = 0.033 and *p* = 0.039) in pediatric patients. No significant differences were found related to other variables (Table 5).

## 4. Discussion

This study analyzed risk factors associated with dental caries disease in children aged 6 to 12 and the severity of caries in this age group. The risk factors involved in the development of caries considered in this work are demographic (age and sex), socioeconomic (educational level of parents, quality of life about oral health), and behavioral (oral hygiene, dietary habits, and physical activity).

In this study, a large percentage (84.1%) of children had at least one caries lesion in mixed dentition. This is important since carious lesions evolve more rapidly in primary teeth than in permanent teeth [5]. Although in Mexico, there has been a decrease in the prevalence of caries over the years, prevalences of up to 88.5% were reported (2020–2021); these results are close to those found in the present investigation [34].

Almost half of the studied population (43.7%) showed at least one severe caries lesion in mixed dentition, which may lead to further oral health problems since active caries lesions are frequently found in children with many carious lesions [35]. An active caries lesion has specific physical characteristics and refers to an ongoing demineralization caused by cariogenic bacteria [11]. Other studies in Mexico reveal more caries lesions in the primary dentition than in the permanent dentition [34].

Regarding the parents’ education, in this study, we found that 31 of them had completed high school, and only 9.9% had a bachelor’s degree or higher. Not only does occupation indicate economic status, educational opportunities, and social hierarchy for both parents and children, but it also provides skills, tools, and knowledge to facilitate the adoption of oral health information [13].

When comparing oral characteristics in the population studied with the parent’s educational level, the latter showed a statistical difference between the presence of fluorosis and the brushing frequency of pediatric patients. Dental fluorosis is caused by excessive exposure to fluoride (more than 1.5 mg/L according to OMS) during odontogenesis and leads to various changes in the development of tooth enamel [36]. The average concentration of fluoride in drinking water in the area is 3.80 mg/L, with a range of 0.1–11.0 mg/L [37]. Fluorosis may be due to a lack of knowledge about the quality of the water consumed by the family or because children swallow fluoridated toothpaste when brushing since children’s toothpaste generally has a pleasant taste.

Epidemiological studies have reported sex-based differences, with a higher prevalence of dental caries in girls than in boys [15]. However, a survey carried out in Tepatitlán, Jalisco, showed a high prevalence of caries without showing a statistical difference according to sex [3].

Girls are more likely to attend dental care services [11]; similarly, in this study, women participated more than men, with 56.3% being female. In Mexico, it was found that males have the highest experience of dental caries (number of teeth affected) compared to females from 5 to 9 years of age [38]. In contrast, this study found that females showed a higher average number of teeth with initial caries lesions. It is also critical to consider that this study’s method of diagnosis of dental caries has been the ICDAS system and not the CPO index since the latter can underestimate the initial lesions in the enamel. This is important because these lesions can progress to moderate and/or severe lesions, cause pain, and require corrective treatment, which is also more costly. It has also been reported that any dental caries experience in early childhood is strongly predictive of dental caries experience in early adolescence [39].

Dental caries experience decreases considerably in both sexes from 10 to 14 years of age due to the exfoliation of the primary dentition [38]. In this study, the mean age of the population was 8.7 ± 1.7, and in all age groups (6–12 years), there are more children with caries than without caries. Notably, none of the children aged 10 showed healthy surfaces (ICDAS 0), maybe because at this age, children have sufficient motor skills to perform effective toothbrushing since the older they are, the better their oral hygiene performance. [40].

In contrast to the above, the number of DMFTs increases with age [19], and following Martignon [11], older children may present caries lesions in advanced stages compared to younger children. For example, Guizar [41] found a relevant association with the increase in and severity of caries in older children in a Mexican population between 3 and 6 years of age. However, this study found a statistical difference (*p* = 0.027) between the age groups (6 to 12 years old) and the presence or absence of caries lesions; future research should consider variables such as dental malposition and the quantification of antibacterial plaque.

In this study, 121 children out of 151 showed a low oral health impact in childhood, translating into a high quality of life related to oral health. Only 30 have an average quality of life related to oral health, either because they have suffered some discomfort due to problems in the oral cavity, such as difficulty sleeping, speaking, chewing, school absenteeism, or the need for emergency corrective treatment.

Comparing oral characteristics in the population studied with the parent’s educational level, the results showed a statistical difference in the brushing frequency of pediatric patients, considering that the habit of proper oral hygiene assisted at an early age is significantly associated with low caries prevalence [12]. In addition, tooth brushing should start from the eruption of the first tooth, and parents are responsible for oral hygiene in childhood to make the removal of bacterial plaque effective since if parents are not involved, oral hygiene in children will not be regular [6]. Having said this, and according to SIVEPAB [38], in children from 6 to 9 years of age, there is a parent–child co-responsibility for oral hygiene. At these ages, there is more variation in oral hygiene.

This study shows a statistical difference between those who brush 0–1 times/day and those who brush two or more times/day (*p* = 0.026). Another similar sample (Venezuelan pediatric population 6–12 years old, using DMFT) found that toothbrushing with fluoridated toothpaste once or more a day had a significant association [9]. Other studies show an association between dental caries and sugar consumption, even in adequate fluoridation [42]. Quantifying the fluoride used in toothbrushing would be interesting since studies find a statistically significant association between health status and toothbrushing with fluoride toothpaste more than once a day [9].

Molar incisor hypomineralization (MIH) refers to an enamel defect involving opacities and sometimes post-eruptive degradation due to enamel porosity, resulting in outcomes ranging from mild atypical caries to severe coronary destruction [43]. In this study, we found 31 (20.5%) pediatric patients with MIH, suggesting that these patients require dental care to avoid future comprehensive health problems associated with this condition. Although the etiology of MIH is unknown, children with health disorders in the first years of life, such as newborn jaundice [44], cesarean-section delivery, and sixth disease (roseola) [45], and those whose mothers underwent illnesses during pregnancy might be more susceptible to MIH [46]. Enamel hypoplasia is a biological factor that predisposes to carriers and is related to deficiencies of vitamin A, D, zinc, iron, and protein energy and malnutrition [42]; vitamin D deficiency is associated with an increased risk of tooth decay due to the relationship that vitamin D has with calcium metabolism and the calcification relationship during the formation of hard tooth tissue [6]. It is worth mentioning that certain foods, such as milk and cheese, have a protective potential against dental demineralization because cow’s milk contains calcium, phosphates, and casein, which have a preventive action against caries [42].

Health problems in early childhood that explain conditions associated with caries and MIH should be investigated since these conditions are caused by demineralization processes and defects in enamel development, respectively.

Regarding physical activity, our study showed that most children (85.2%) have deficient physical activity habits, which could represent future health problems; in contrast, young people who exercise regularly do so because it gives them a huge sense of well-being [47]. In addition, caries and oral health prevalence in adolescents who regularly exercise are significantly better than those who do not, and they need less restorative dental treatment [20].

Primary caregivers, family members, and/or nursery and daycare personnel should also provide nutritional health education. They should contribute to developing good eating habits in children [12], just as it should be in educating them about oral care habits for proper oral health since caries share risk factors with prevalent high-prevalence diseases such as obesity and diabetes [11]. In this study, the frequency of food consumed between meals was considered. Although most had deficient eating habits, no significant differences were found with those with adequate eating habits. Bearing in mind that the children’s diet is the parent’s responsibility, the child will establish their dietary habits for life, hence the taste for early consumption of sugar in food that can increase the risk of tooth decay and childhood obesity [22].

Although a high prevalence of caries has been reported in overweight or obese children [34], in our work, we did not find a significant difference between caries severity and overweight or obesity. Therefore, future research could include nutritional variables involving the consumption of specific foods and nutrients that predict some protection against the development of caries lesions since nutrition is the main factor in inflammation and infection, as it affects the innate and adaptive defense response and induces infectious diseases of the oral mucosa, caries, and periodontal disease [42].

Due to the complexity of the caries process, future research could link various factors that influence this process, including aspects of preventive dentistry, previous treatment, parental habits, maternal oral health, problems during pregnancy, cultural and economic aspects, general health, developmental defects, use of medication, periodontal disease, malocclusion, and specific biological factors such as quantification of salivary proteins and genetic variants.

Following on from the above, several individuals with the same protective factors (fluoridated water consumption, favorable caries habits, and good hygiene habits) present different caries patterns, which genetic factors can explain since some genes are related to the incidence of dental caries (enamel formation and salivary composition) [48].

This study has limitations since the sample was drawn from a population of 6–12-year-old children who attend the Comprehensive Medical Care Center and because patients who come to the center regularly require restorative treatment for dental problems such as pain and inflammation of soft tissues. The results are valid only for the population studied, and caution should be exercised as they are not representative of the entire population of children. However, they are part of the oral health characteristics of Los Altos, Jalisco population.

The information provided by our research is essential to better understand the oral health problems afflicting our child population. It includes knowledge about which health institutions can carry out prevention and oral health care programs and that health authorities should implement policies that favor adequate oral health in children. It is essential to mention schools, where parents and children can be made aware of the importance of improving and adopting good oral hygiene, physical activity, and eating habits, which will prevent them from suffering from being overweight, obesity, and tooth decay.

## 5. Conclusions

The analysis in the present study indicated a deficiency in dietary habits and a considerable prevalence of dental caries among children aged 6–12. Caries was linked to age and tooth brushing practices. It is convenient to identify specific nutrition, brushing, and oral hygiene practices in order to reinforce techniques, better understand the health problem, and provide updated knowledge with which health institutions can also implement oral health prevention programs that also follow up and apply policies that verify practical skills that allow schools, children, and parents to have theoretical knowledge and raise awareness to adopt good oral hygiene and eating habits.

Females had a higher average number of teeth with initial carious lesions, and parental education was associated with both the presence of fluorosis and the likelihood of regular tooth brushing. Future research could explain why pediatric populations exposed to the same risk factors develop caries and others do not since a risk factor is not studied as an isolated entity but as a part of the complex network of factors involved in the development of caries.

We also identified a lack of attention to oral health problems in this group of children and a need for preventive and corrective dental treatment. The results help develop a project based on pediatric dentists’ intervention strategies and prevention programs that could bet on early detection and thus prevent related problems such as unsightly lesions, irreversible recovery, and sleeping or eating problems, in addition to avoiding time-consuming, costly, and/or painful treatments for the vulnerable child population.

## Figures and Tables

**Table 1:** Descriptive data on sociodemographic and other variables in the study group.

Sociodemographic and Other Characteristics	Categories	Frequency	Percentage
Sex	Female	85	56.3
	Male	66	43.7
Age (years)	6	20	13.2
	7	19	12.6
	8	27	17.9
	9	33	21.9
	10	20	13.2
	11	26	17.2
	12	6	4.0
Obesity	Healthy	115	76.2
	Obesity	36	23.8
ECOHIS ^a^	Low	121	80.1
	Medium	30	19.9
Quality of life in oral health	Medium	30	19.9
	High	121	80.1
Parent’s education	Primary	29	19.2
	Secondary	76	50.3
	High School	31	20.5
	University	15	9.9
Eating habits	Adequate	22	14.8
	Deficient	127	85.2
Caries severity	Healthy	24	15.9
	Initial	26	17.2
	Moderate	35	23.2
	Severe	66	43.7
Caries	Absent	24	15.9
	Present	127	84.1
MIH ^b^	Absent	120	79.5
	Present	31	20.5
Fluorosis	Absent	142	94.0
	Present	9	6.0
Toothbrushing (times/day)	0–1	82	54.3
	2 or more	69	45.7
Dental floss	Yes	6	4.0
	No	145	96.0
Teeth affected by caries (median ± SE)	Initial	1.709	2.042
	Moderate	0.967	1.174
	Severe	1.457	2.579

^a^ Early Child Oral Health Impact Scale; ^b^ Molar incisor hypomineralization.

**Table 2:** Comparison of sociodemographic data and other characteristics according to the presence of caries.

Sociodemographic and Other Characteristics	Categories	Healthy *n* (%)	Caries *n* (%)	*p*	OR (95% CI)
Sex	Female	14 (16.5)	71(83.5)	0.826	1.10 (0.45–2.67)
	Male	10 (15.2)	56 (84.8)		
Age	6 years	3 (15.0)	17 (85.0)	0.027	NA
	7 years	5 (26.3)	14 (73.7)		
	8 years	7 (25.9)	20(74.1)		
	9 years	4 (12.1)	29 (87.9)		
	10 years	0 (0.0)	20 (100.0)		
	11 years	2 (7.7)	24 (92.3)		
	12 years	3 (50.0)	5 (50.0)		
Obesity	Healthy	20 (17.4)	95 (82.6)	0.368	1.68 (0.53–5.30)
	Obesity	4 (11.1)	32 (88.9)		
ECOHIS ^a^	Low	20 (16.5)	101 (83.5)	0.668	1.29 (0.40–4.09)
	Medium	4 (13.3)	26(86.7)		
Quality of life in oral health	Medium	4 (13.3)	26 (86.7)	0.668	1.29 (0.40–4.09)
	High	20 (16.5)	101 (83.5)		
Parent’s education	Primary	5 (17.2)	24 (82.8)	0.524	NA
	Secondary	12 (15.8)	64 (84.2)		
	High School	3 (9.7)	28 (90.3)		
	University	4 (26.7)	11 (73.3)		
Eating habits	Adequate	1 (4.5)	21 (95.5)	0.110	4.64 (0.59–63.3)
	Deficient	23 (18.1)	104(81.9)		
Toothbrushing (times/day)	0–1	18 (22.0)	64 (78.0)	0.026	2.95 (1.10–7.93)
	2 or more	6 (8.7)	63 (91.3)		
Dental floss	Yes	1 (16.7)	5 (83.3)	0.958	1.06 (0.11–9.5)
	No	23 (15.9)	122 (84.1)		

^a^ Early Child Oral Health Impact Scale. OR: odds ratio. NA: not applicable.

**Table 3:** Comparison of sociodemographic data and other characteristics according to caries severity.

Sociodemographic and Other Characteristics	Category	Caries Severity				*p*
		Healthy *n* (%)	Initial *n* (%)	Moderate *n* (%)	Severe *n* (%)	
Sex	Female	14 (16.5)	19 (22.4)	17 (20.0)	35 (41.2)	0.246
	Male	10 (15.2)	7 (10.6)	18 (27.3)	31 (47.0)	
Age (years)	6	3 (15.0)	4 (20.0)	3 (15.0)	10 (50.0)	0.159
	7	5 (26.3)	4 (21.1)	3 (15.8)	7 (36.8)	
	8	7 (25.9)	1 (3.7)	6 (22.2)	13 (48.1)	
	9	4 (12.1)	5 (15.2)	8 (24.2)	16 (48.5)	
	10	0 (0.0)	7 (35.0)	6 (30.0)	7 (35.0)	
	11	2 (7.7)	5 (19.2)	7 (26.9)	12 (46.2)	
	12	3 (50.0)	0 (0.0)	2 (33.3)	1 (16.7)	
Obesity	Healthy	20 (17.4)	21 (18.3)	26(22.6)	48 (41.7)	0.687
	Obesity	4 (11.1)	5 (13.9)	9 (25.0)	18 (50.0)	
ECOHIS ^a^	Low	20 (16.5)	21 (17.4)	29 (24.0)	51 (42.1)	0.882
	Medium	4 (13.3)	5 (16.7)	6 (20.0)	15 (50.0)	
Quality of life in oral health	Medium	4 (13.3)	5 (16.7)	6 (20.0)	15 (50.0)	0.882
	High	20 (16.5)	21 (17.4)	29 (24.0)	51 (42.1)	
Parent’s education	Primary	5 (17.2)	4 (13.8)	4 (13.8)	16 (55.2)	0.142
	Secondary	12 (15.8)	10 (13.2)	18 (23.7)	36 (47.4)	
	High School	3 (9.7)	10 (32.3)	7 (22.6)	11 (35.5)	
	University	4 (26.7)	2 (13.3)	6 (40.0)	3 (20.0)	
Eating habits	Adequate	1 (4.5)	5 (22.7)	5 (22.7)	11 (50.0)	0.432
	Deficient	23 (18.1)	21 (16.5)	28 (22.0)	55 (43.3	
Toothbrushing (times/day)	0–1	18 (22.0)	13 (15.9)	19 (23.2)	32 (39.0)	0.155
	2 or more	6 (8.7)	13 (18.8)	16 (23.2)	34 (49.3)	
Dental floss	Yes	1 (16.7)	1 (16.7)	1 (16.7)	3 (50.0)	0.982
	No	23 (15.9)	25 (17.2)	34 (23.4)	63 (43.4)	

^a^ Early Child Oral Health Impact Scale.

**Table 4:** Comparison of caries characteristics according to sex.

Caries Status Median (IQR)	Female *n* = 85	Male *n* = 66	*p*
Initial	1.94 (0.00–3.00)	1.40 (0.00–2.00)	0.039
Moderate	0.94 (0.00–2.00)	1.00 (0.00–2.00)	0.489
Severe	1.25 (0.00–1.00)	1.71 (0.00–2.00)	0.300
Active	2.22 (0.00–4.00)	2.63 (0.00–4.00)	0.580
Inactive	1.89 (0.00–3.00)	1.54 (0.00–2.75)	0.154

IQR: interquartile range.

**Table 5:** Comparison of oral characteristics according to parent’s educational level.

Sociodemographic and Other Characteristics	Category	Primary *n* (%)	Secondary *n* (%)	High School *n* (%)	University *n* (%)	*p*
Caries	Absent	24 (18.9)	64 (50.4)	28 (22.0)	11 (8.7)	0.509
	Present	5 (20.8)	12 (50.0)	3 (12.5)	4 (16.7)	
MIH ^a^	Absent	24 (20.0)	60 (50.0)	24 (20.0)	12 (10.0)	0.979
	Present	5 (16.1)	16 (51.6)	7 (22.6)	3 (9.7)	
Fluorosis	Absent	29 (20.4)	73 (51.4)	28 (19.7)	12 (8.5)	0.033
	Present	0 (0.0)	3 (33.3)	3 (33.3)	3 (33.3)	
Toothbrushing	0–1	15 (18.3)	36 (43.9)	18 (22.0)	13 (15.9)	0.039
	2 or more	14 (20.3)	40 (58.0)	13 (18.8)	2 (2.9)	
Dental floss	Yes	1 (16.7)	2 (33.3)	2 (33.3)	1 (16.7)	0.656
	No	28 (19.3)	74 (51.0)	29 (20.0)	14 (9.7)	

^a^ Molar incisor hypomineralization.

## Data Availability

The raw data supporting the conclusions of this article will be made available by the authors on request.
